# SLEEP IN LATE LIFE: RECENT RESEARCH ON PSYCHIATRIC CORRELATES AND TREATMENT

**DOI:** 10.1093/geroni/igz038.171

**Published:** 2019-11-08

**Authors:** Mary E Dozier

**Affiliations:** South Texas Veterans Health Care System, San Antonio, Texas, United States

## Abstract

Sleep is an often overlooked health factor, particularly in older adults. Sleep disturbance is associated with increased functional impairment as well as poorer cognitive, mental, and physical health trajectories. Understanding the clinical impact of disturbed sleep, and the optimal targets for intervention, is critical for the promotion of health and well-being in older adults. This symposium will highlight recent findings that advance the extant knowledge on the interplay of sleep disturbance and physical and psychiatric co-morbidities in older adults across a variety of settings. Darina V. Petrovsky will discuss the impact of medical, demographic, and contextual factors on excessive daytime sleepiness in older adults receiving long-term services and supports. Kathi L. Heffner will present data on a recent study examining change in slow wave sleep, and subsequent change in osteoarthritis pain, following insomnia treatment. Courtney Bolstad will discuss the differential impact of onset, maintenance, and terminal insomnia on anxiety and depression symptoms in community-dwelling older adults. Eliza Davidson will present research on the association between sleep disturbance and hoarding symptoms in older adults engaged in behavioral interventions for hoarding disorder. Finally, Christina McCrae will discuss the relationship between sleep and cognition in older adults with insomnia.

